# Volumetric high-resolution X-ray phase-contrast virtual histology of breast specimens with a compact laboratory system

**DOI:** 10.1109/TMI.2021.3137964

**Published:** 2022-05-02

**Authors:** Lorenzo Massimi, Tamara Suaris, Charlotte K. Hagen, Marco Endrizzi, Peter R. T. Munro, Glafkos Havariyoun, P.M. Sam Hawker, Bennie Smit, Alberto Astolfo, Oliver J. Larkin, Richard M. Waltham, Zoheb Shah, Stephen W. Duffy, Rachel L. Nelan, Anthony Peel, J. Louise Jones, Ian G. Haig, David Bate, Alessandro Olivo

**Affiliations:** Department of Medical Physics and Biomedical Engineering, University College London, London, UK; St Bartholomew's Hospital, Barts Health NHS Trust, West Smithfields, London, UK; Department of Medical Physics and Biomedical Engineering, University College London, London, UK; Department of Medical Physics and Biomedical Engineering, University College London, London, UK; Department of Medical Physics and Biomedical Engineering, University College London, London, UK; Department of Medical Physics and Biomedical Engineering, University College London, London, UK; Nikon X-Tek Systems, Tring Business Centre, Icknield Way, Tring, Hertfordshire, UK; Nikon X-Tek Systems, Tring Business Centre, Icknield Way, Tring, Hertfordshire, UK; Nikon X-Tek Systems, Tring Business Centre, Icknield Way, Tring, Hertfordshire, UK; Nikon X-Tek Systems, Tring Business Centre, Icknield Way, Tring, Hertfordshire, UK; Nikon X-Tek Systems, Tring Business Centre, Icknield Way, Tring, Hertfordshire, UK; Barts and the London School of Medicine and Dentistry, Queen Mary University of London, Newark St, London, UK; Barts and the London School of Medicine and Dentistry, Queen Mary University of London, Newark St, London, UK; Barts and the London School of Medicine and Dentistry, Queen Mary University of London, Newark St, London, UK; St Bartholomew's Hospital, Barts Health NHS Trust, West Smithfields, London, UK; St Bartholomew's Hospital, Barts Health NHS Trust, West Smithfields, London, UK; Barts and the London School of Medicine and Dentistry, Queen Mary University of London, Newark St, London, UK; Nikon X-Tek Systems, Tring Business Centre, Icknield Way, Tring, Hertfordshire, UK; Nikon X-Tek Systems, Tring Business Centre, Icknield Way, Tring, Hertfordshire, UK; Department of Medical Physics and Biomedical Engineering, University College London, London, UK

**Keywords:** Breast conserving surgery, multi-resolution imaging, X-ray phase contrast

## Abstract

The assessment of margin involvement is a fundamental task in breast conserving surgery to prevent recurrences and reoperations. It is usually performed through histology, which makes the process time consuming and can prevent the complete volumetric analysis of large specimens. X-ray phase contrast tomography combines high resolution, sufficient penetration depth and high soft tissue contrast, and can therefore provide a potential solution to this problem. In this work, we used a high-resolution implementation of the edge illumination X-ray phase contrast tomography based on “pixel-skipping” X-ray masks and sample dithering, to provide high definition virtual slices of breast specimens. The scanner was originally designed for intra-operative applications in which short scanning times were prioritised over spatial resolution; however, thanks to the versatility of edge illumination, high-resolution capabilities can be obtained with the same system simply by swapping x-ray masks without this imposing a reduction in the available field of view. This makes possible an improved visibility of fine tissue strands, enabling a direct comparison of selected CT slices with histology, and providing a tool to identify suspect features in large specimens before slicing. Combined with our previous results on fast specimen scanning, this works paves the way for the design of a multi-resolution EI scanner providing intra-operative capabilities as well as serving as a digital pathology system.

Breast cancer is the most common cancer in women in the UK, and it accounts for approximately 15% of all the diagnosed cancers in the country [1]. The achievement of clear margins in resected specimens is one of the key objectives in breast conserving surgery. While the removal of an excessive amount of tissue can lead to a poor cosmetic outcome, the presence of cancer at the margins of the excised tissue can lead to recurrences and re-operations, with significant stress for the patients and increased costs for the healthcare service. The margin assessment is usually performed through histology both intra and post-operatively [2], [3]. However, the histological assessment of an entire breast excision is time consuming and requires extensive sample preparation. Therefore, the availability of a volumetric technique capable of providing histology-like images over the entire specimen is highly desirable. Conventional techniques such as optical microscopy or spectroscopy provide volumetric information but have, in general, a reduced penetration depth, hindering their application to large specimens such as large wide local excisions or mastectomies [4], [5]. On the other hand, while X-rays are one of the methods of choice for breast cancer screening, their application to intra and post-operative assessment of breast specimens is still the subject of investigations [6], [7]. The main limitation is that, while conventional X-ray imaging provides sufficient penetration depth and good spatial resolution, it provides limited soft tissue contrast if staining agents are not used, which affects its application to biomedical imaging. The low soft tissue contrast represents an intrinsic limitation of conventional x-rays, which can be overcome only by changing the image formation mechanism [8] for example by x-ray phase contrast imaging (XPCI).

XPCI exploits the phase shift suffered by X-rays when passing through a specimen instead of conventional attenuation, and was shown to provide soft tissue contrast down to cellular level also with minimal sample preparation [9]–[11]. Combined with the volumetric capability of tomography, XPCI allows the virtual 3D analysis of entire specimens, opening the way to quantitative digital pathology [12]–[14]. For this reason, X-ray phase contrast tomography (XPCT) has been extensively used to image breast specimens at synchrotron radiation facilities [15]–[17]. However, the need for large-scale facilities prevented a wide application of this technology to healthcare. Therefore, there is a large interest in breast imaging using laboratory based phase contrast techniques [18]–[20]. Recently, a first pre-commercial prototype of an edge-illumination (EI) XPCI scanner specifically designed for intra-operative breast imaging has been presented, achieving a scanning time of 10 minutes at 100 *μ*m spatial resolution over a large field of view [7]. EI is based on the use of two absorption gratings, usually referred to as masks, the design of which can be tailored to specific imaging needs. In this work, we exploit this flexibility to extend the capabilities of the prototype, by adding a multi-resolution acquisition mode so that the scanner can also be used as a 3D virtual histology system. This has been possible thanks to the combination of a dedicated mask set and of the digital recombination of multiple frames acquired while the sample is laterally displaced in sub-pixel steps, achieving a final resolution comparable to the size of the apertures in the pre-sample mask. We quantitatively compared this high-resolution working mode to the previous intra-operative one, and we investigated its use for digital histology by imaging tumour-bearing breast specimens with both modes. We found that the high-resolution mode allows for a better visualization of several key features for the post-operative assessment of breast specimens, such as fine tissue strands and clusters of immune and cancer cells, as validated through a comparison with conventional hematoxylin and eosin (H&E) histology.

## I Results

**Fig. 1:**
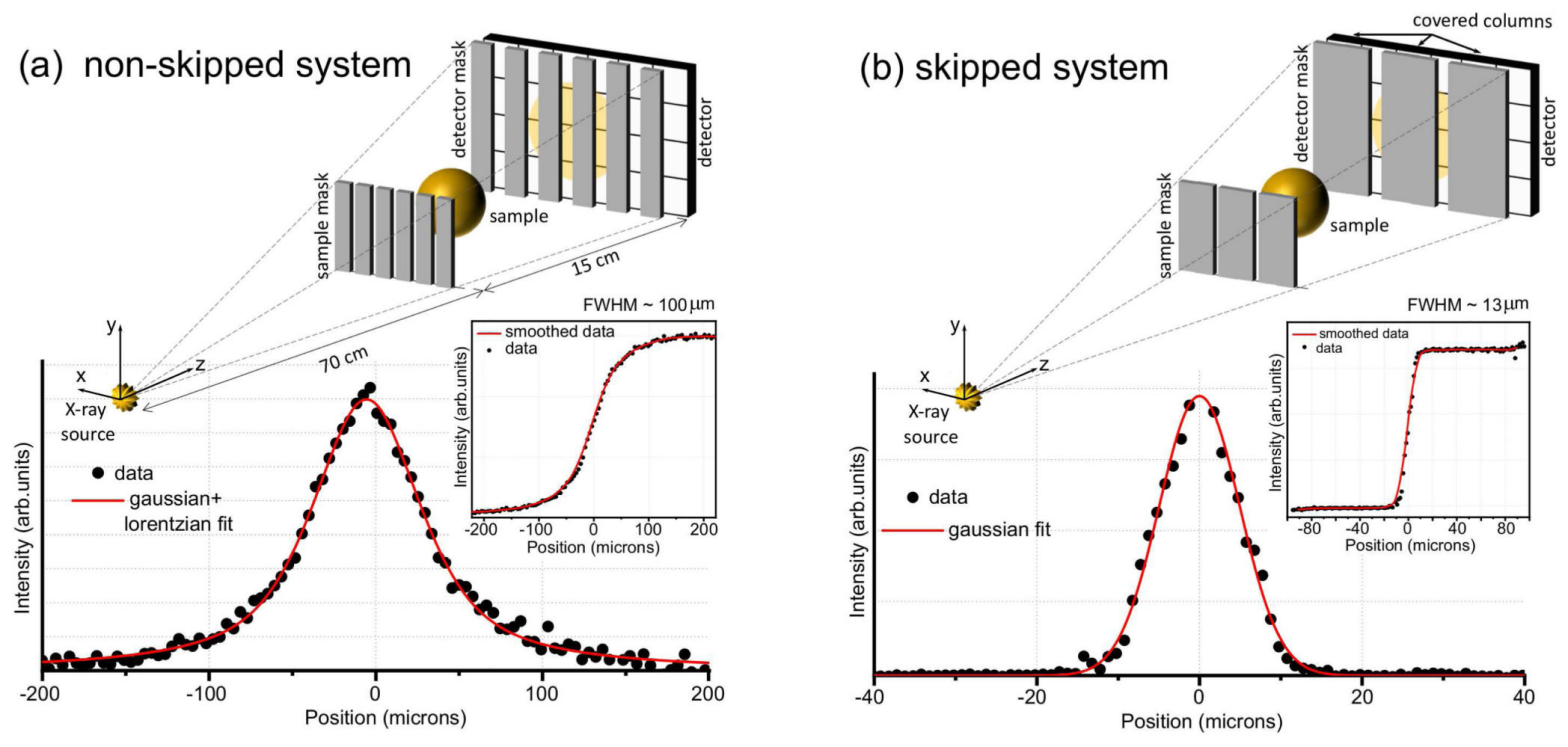
Panel (a) and (b) show the experimental LSF and a schematic view of the corresponding EI system for the non-skipped and skipped mask configurations, respectively. For both panels, the corresponding experimental and smoothed ESF (the derivative of which yields the LSF) is also reported in the insets. A fit of both LSF profiles is also shown. In the skipped mask case, 8 dithering steps have been acquired and recombined prior to the ESF estimation.

To understand the impact of skipped and non-skipped masks on the system resolution, an analysis of the Line Spread Function (LSF) at the sample position is reported in Fig.1, alongside a scheme of the corresponding edge illumination setup. In particular, Fig.1(a) and (b) show the LSF acquired by using the non-skipped and skipped configurations, respectively. In addition, the smoothed edge spread function (ESF) is shown in both cases in the insets, alongside the corresponding raw experimental ESF data. When the non-skipped masks are used (Fig.1(a)), the LSF is well fitted by a linear combination of Gaussian and Lorentzian profiles, with the latter taking into account the long tails. The value of the LSF's Full Width at Half Maximum (FWHM) has been considered as the system resolution. A good match was observed between the fitted profile and experimental data, indicating a spatial resolution FWHM_*ns*_ = 100 *μ*m which is about twice the pixel size. This is in agreement with the detector LSF measured without the masks, showing that in this case the system resolution is detector-driven and limited by the cross-talk between pixels [21]. The use of skipped masks addresses this limitation by reducing the cross-talk between adjacent columns, as shown by the corresponding LSF in Fig.1(b). In this case, the long tails of the LSF are eliminated and a Gaussian profile provides a good model, resulting in a system resolution FWHM_*s*_ = 13 *μ*m which is very close to the theoretically expected value of 12 *μ*m given by the aperture size [22].

**Fig. 2:**
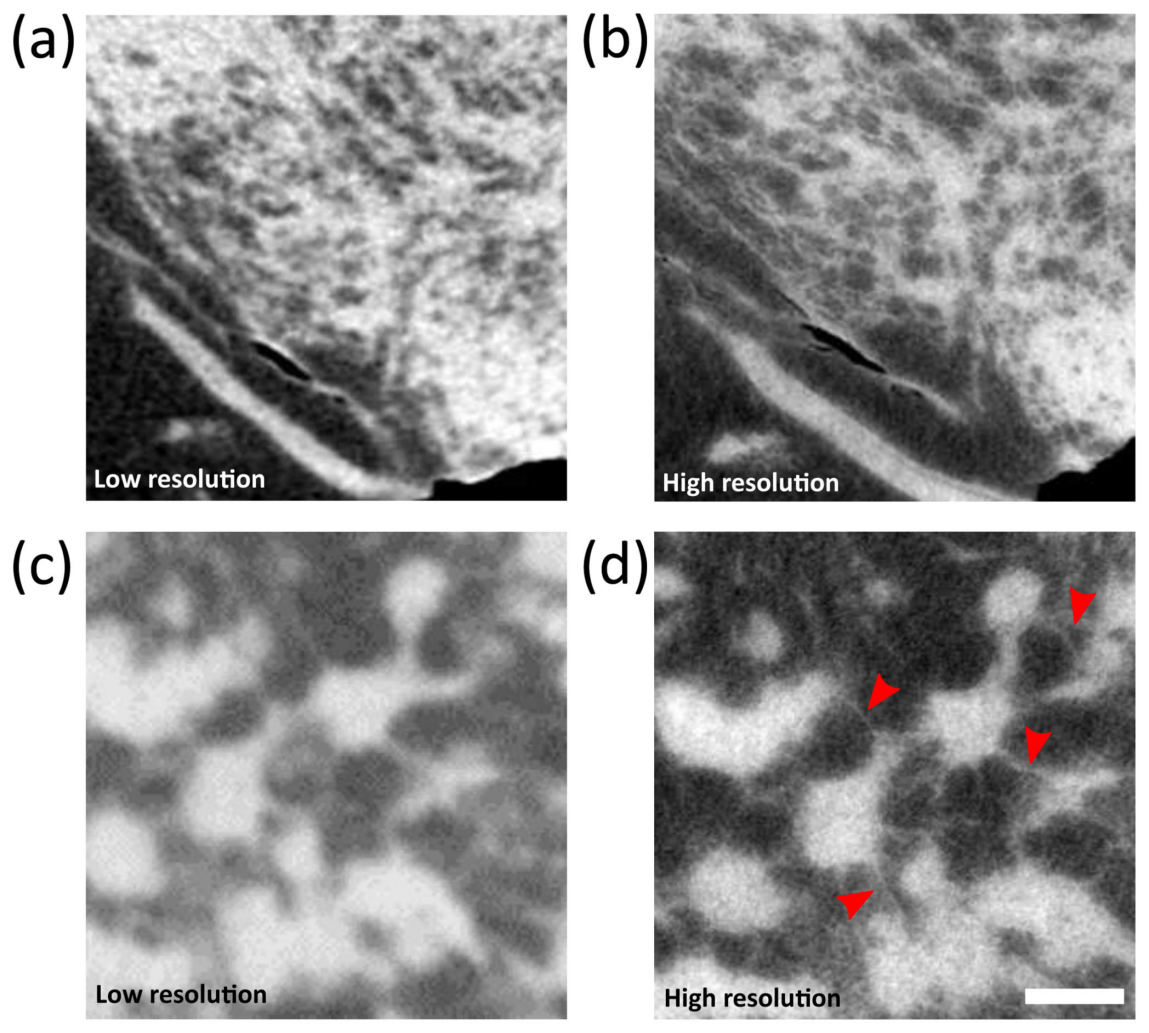
Panel (a), (c) and (b), (d) show a CT slice from two specimens acquired with both low resolution and high resolution configurations, respectively. Red arrows in panel (d) point at fine features invisible or barely visible in the low resolution scan. Scale bar is 1 mm for all the panels.

A comparison between CT slices of the same samples obtained with skipped masks and 8 dithering steps (high-resolution) and with the non-skipped masks and no dithering (low-resolution) is shown in Fig.2. While more dithering steps can be acquired, the recombination of 8 steps provides a spatial sampling (12.5 *μ*m) comparable to the aperture size, which represents the achievable resolution limit, while maintaining a reasonable scanning time [22]. In the first example shown in Fig.2(a) the increase in resolution can be easily appreciated by looking at the improved details of the complex texture observed in the fibro-glandular tissue. A higher definition is apparent when the high-resolution mode is used (panel(b)). The increased resolution allows to identify fine tissue structures invisible in the low-resolution scans, as shown in the second specimen in Fig.2(c). In particular, high-resolution in panel (d) shows fine tissue strands connecting lobules (highlighted by red arrows), which are barely visible or completely invisible in the low-resolution image on panel (c).

**Fig. 3:**
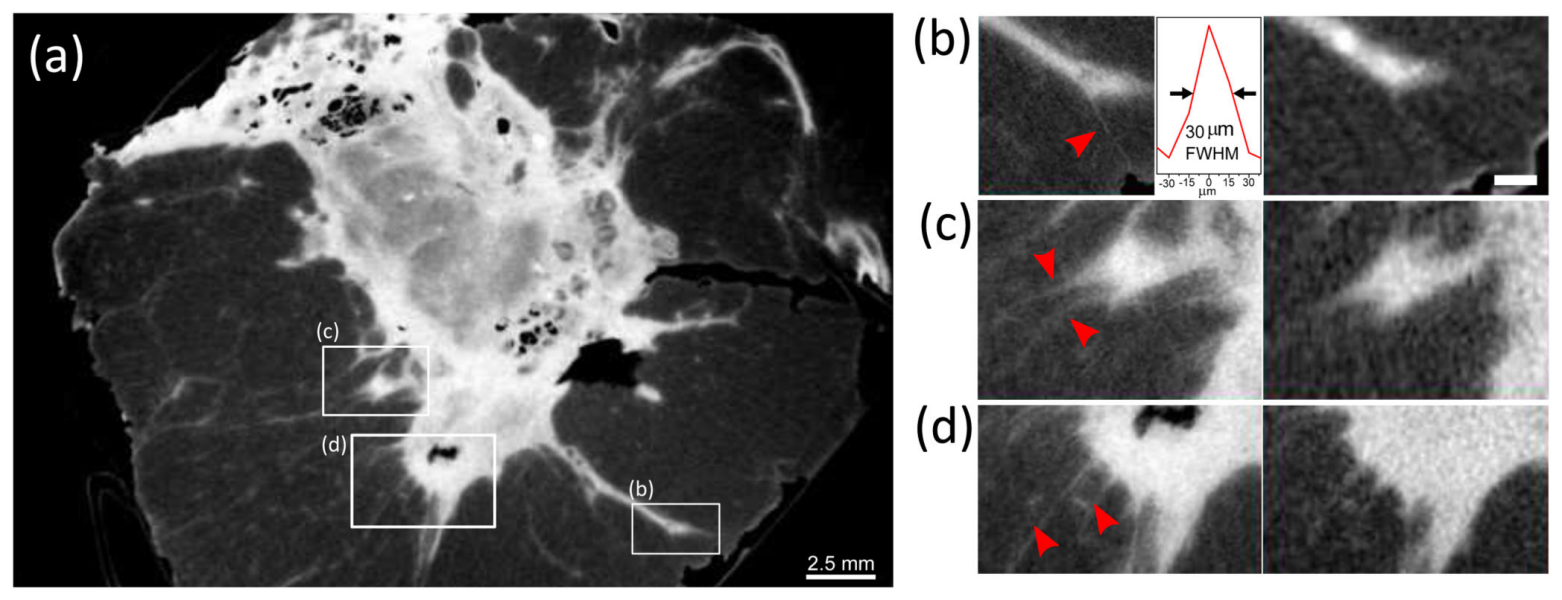
Panel (a) shows a CT slice of a fixed breast specimen obtained with the skipped sample mask. Panel (b) to (c) show a comparison between ROIs extracted from the high-resolution CT scan obtained with skipped sample masks (left hand side) and the low-resolution scan acquired with the non-skipped sample mask (right hand side). Red arrows in the high-resolution ROIs points at fine strands of tissue that are invisible in the low-resolution scan. Scale bar is 500 *μ*m.

Another example focused on the improved visibility of tissue strands is shown in Fig.3. In particular, panels from (b) to (d) show zoomed-in views of the regions highlighted in panel (a) for both high- and low-resolution scans (left and right, respectively). Panel (b) shows a fine tissue strand departing from a main fibro-glandular structure close to the edge of the specimen (see red arrowhead). Remarkably, the thicker end to the left-hand side of this strand can be seen also in the low resolution image, but the remaining part propagating towards the edge of the specimen is completely invisible. A width of only 30 *μ*m, which is approximately twice the estimated spatial resolution, is measured for this strand as illustrated in the inset. This is an important result from a diagnostic point of view, since similar fine structures can be related to tumour infiltration, and could therefore be representative of very difficult to detect cases of incomplete tumour excision during surgery. Similar results are shown in Fig.3(c) and (d), where tissue strands at the edge of the fibro-glandular region are clearly detected in the high-resolution scan, but barely visible in low-resolution one.

**Fig. 4:**
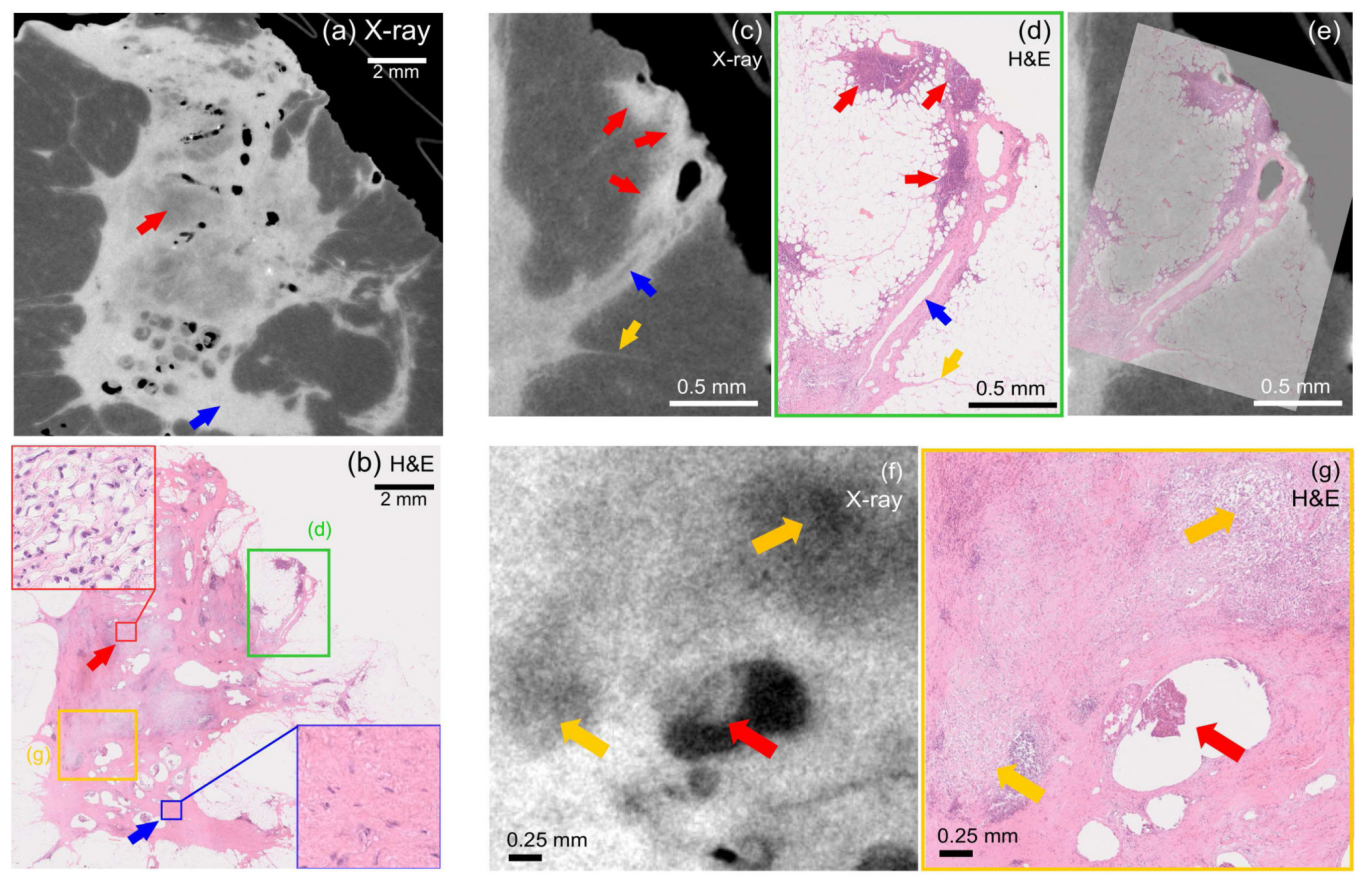
Comparison between XPCT and H&E staining. Panels (a) and (b) show matched high-resolution CT and histology slices, respectively. Red and blue arrows in panel (a) point at regions with different density as observed in the CT slice, with the zoomed-in regions in panel (b) highlighting the different cellular structure of the same areas. Panels (c) and (d) show a zoomed in comparison between XPCT and histology slices. Red, blue and yellow arrows point at clusters of immune cells, a milk duct and a thin strand of tissue, respectively. XPCT and histology slices are overlapped in panel (e). A similar comparison is reported in panels (f) and (g) where yellow arrows point at areas of response to chemotherapy, which appear with a lower density. The red arrow points at a region of a residual infiltrating ductal carcinoma nodule.

The number of details visible in the high-resolution CT slices allows a good correlation with the histological sections. This point is illustrated in Fig.4, where a comparison between both techniques is reported. Regions with different densities are highlighted in panel (a) by red and blue arrows. This difference in density is due to a different composition of the tissue at the cellular level, as revealed by histology and shown by the zoomed up regions in Fig.4(b), framed in red and blue for ease of comparison. In particular, the region with lower density (red arrow in panel (a)) is referred to as tumour bed, which is an area of loose connective tissue with associated inflammatory infiltrate, typical of areas of response to chemotherapy as shown by histology. The presence of a lower density of connective tissue within these areas leads to the lower density observed in the CT slice. The difference in voxel values between this region and the surrounding tissue is sufficient to allow an intensity-threshold segmentation (see supplementary materials). On the other hand, the region with higher density (blue arrow in panel (a)) shows a compact fibrous tissue with lower cellular density corresponding to healthy tissue. A similar comparison is illustrated for a zoomed-in region in Fig.4(c) to (e). Comparing the XPCT and the histology slices, similar details are visible. In particular, clusters of high cellular density recognized as lymphocytes, which may be connected to the level of immune response to therapy, are indicated by the red arrows at the edge of specimen in panels (c) and (d), close to a section of a milk duct indicated the blue arrow. The yellow arrow points at a thin fibrous strand observed both in the X-ray image and the histological slice. Finally, Fig.4(e) shows the overlap between the CT slice and the corresponding histological section, further demonstrating the good match between the details observed in both. A further comparison reported in 4(f) and (g) focuses on a region of a residual infiltrating ductal carcinoma nodule within artefactual tissue spaces, indicated by the red arrow in both panels. In addition, two lower density regions are also observed in the XPCT slice (see yellow arrows), and perfectly matched with the corresponding loose connective tissue regions in the histology.

## Discussion

Compact systems based on EI XPCI are showing potential for medical applications. Recently, it has been shown that a low-resolution EI system provides diagnostic advantages in breast surgery with a scanning time compatible with the intra-operative use [7], [23]. Such a constraint on scanning time imposed a compromise on resolution. When scanning time is not a constraint, the same system allows for the acquisition of higher resolution scans, through combining dithered acquisition and skipped masks [22]. Dithered acquisitions provide better spatial sampling and increase the range of high frequencies in the image, otherwise lost because of aliasing. The ultimate spatial resolution that can be achieved in this way is in the range of the mask aperture size, which for this work was 12 *μ*m, and is independent from both detector pixel size and x-ray source focal spot size. Dithering is effective only if the system cross-talk is minimal. When non-skipped masks are used in combination with indirect conversion detectors, the use of dithering creates artefacts that reversely affect image resolution if no further processing is carried out [24], [25]. However, dithered acquisitions translate into a significant increase in the total scanning time. Compared to low resolution CT scans, the scanning time for the high-resolution ones is increased by a factor at least equal to the number of dithering steps. We demonstrated that the increased spatial resolution provides a much better visibility of fine tissue strands, which is particularly valuable since spiculated features are one of the main hallmarks of malignancy in breast lesions. The ability to visualize fine tissue strands in large breast specimens, and the similarity between XPCT slices and histological sections, suggests that high resolution EI could provide a valuable aid for pathologists. For example, it would allow localising suspicious features in advance, therefore guiding and optimising subsequent histopathology workup as already demonstrated for other types of tissue [26], [27]. In particular, it may help in the identification of fine strands of tissue that may reveal that the tumour extends beyond what was observed with e.g. conventional pre-surgical imaging, and therefore prompt the histological analysis of areas of tissue that would otherwise be ignored. The ability to detect tissue response to chemotherapy may enable studies on the effect of various anti-cancer drugs. Other studies have highlighted the advantages of high-resolution, 3D “digital histology” images in term of e.g. providing a full 3D context to the observed features, and eliminating the artifacts arising from the need to cut the tissue [14]. All this notwithstanding, it is important to note that, while various tumour related features have been made visible by the use of our system, further investigation is needed to understand the specific clinical relevance of these images.

It is also worth noting that smaller apertures sizes are well within the reach of current fabrication methods, potentially providing higher resolution levels if required by a specific virtual histology application.We did not observe a significant shrinkage after wax embedding, minimally affecting the ability of a pathologist to slice the breast tissue specimens based on guidance from high-resolution 3D images obtained with the proposed system (see supplemental information). While in this work we used small samples, the field of view is limited only by the mask size, which in principle allows covering also full mastectomy samples with the same spatial resolution; masks as large as 12 × 12 cm^2^ have already been fabricated [7], which is still below current fabrication limits. The use of two different sets of masks required user intervention to switch between low- and high-resolution modes, with consequent need for system realignment. However, we have recently shown that skipped and non-skipped designs can be simultaneously arranged on the same mask [28]. This allows for the design of a multi-purpose CT scan: such a machine could be used for both low-resolution intra-operative applications and for high-resolution virtual histology, simply by laterally displacement of a mask featuring both designs. If still needed, a fine adjustment can then be achieved through automatic procedures, thus minimizing the need for any user intervention making this device suitable for clinical working theatres [29].

## Conclusions

We showed how an EI XPCT system previously developed for intra-operative imaging can be adapted to serve as a digital pathology device thanks to the increase in spatial resolution obtained through the combination of a skipped mask design and dithered acquisitions. This ultimately leads to a resolution close to the theoretical limit determined by the size of the apertures in the sample mask, while preserving the large field of view achievable through the use of large masks. We have also shown that such an enhanced resolution provides improved visibility of several fine tissue details, included tumour-related ones, that may help pathologists identify features of interest in the whole volume, to then carry out further investigations through e.g. histological slicing, while also being able to put the extracted information in a 3D context.

## II Methods

### Edge illumination

EI is an X-ray phase detection scheme specifically designed to work with multi-energy and divergent beams from conventional X-ray sources [30], [31]. A schematic view of a conventional (non-skipped) EI system is shown in Fig.1(a). It is based on the use of two absorption gratings, usually referred to as masks. The first mask is placed before the sample while the latter is in front of the detector. The first mask, usually referred to as the sample mask, shapes the beam into a series of beamlets. The convolution between the beamlet intensity profiles and the detector mask apertures is referred to as the illumination curve (IC), and it can be mathematically described as:

(1) $$IC\left(x\right)=\left(A_{1}*S*A_{2}\right)\left(x\right)$$

where * denotes convolution, *A*_1_ and *A*_2_ are the sample and detector masks profiles and S is the source profile. When passing through a specimen, each beamlet is attenuated and/or re-fracted according to its composition and thickness. The effect of the sample on each beamlet is described by the sample and energy dependent *β* (*x, y, z, E*) and *δ* (*x, y, x, E*) coefficients for transmission and refraction, respectively. Therefore, for each pixel (x,y) it is possible to write:

(2) $$I\left(x,y\right)=I_{0}T\left(x,y\right)IC\left(x-\Delta x\right)$$

where *T*(*x, y*) is the transmitted intensity described by the Beer-Lambert law $T\left(x,y\right)=e^{-\int_{0}^{d}\beta \left(x,y,z\right)dz}$, and Δ*x* ~ *z_od_*Δ*θ* expresses the shift of each beamlet, with *z_od_* the object to detector distance and Δ*θ* the refraction angle expressed as $\Delta \theta \left(x,y\right)=\nabla_{x,y}\int_{0}^{d}\delta \left(x,y,z\right)dz.$ In both cases the integral is taken across the sample thickness *d*, and energy dependence has been neglected for simplicity. The second mask, usually referred to as the detector mask, provides sensitivity to the refraction angle by creating insensitive regions between pixels: a decrease or increase of intensity is measured for each pixel if the beamlet is refracted away or towards it, respectively [30]. While conventional absorption can be obtained from a single intensity measurement, the extraction of the refraction signal requires a pixel-wise evaluation of the shift in the position of each beamlet. Therefore, it requires the acquisition of more than one image with the masks being displaced between acquisitions to sample the beamlet intensity profile. In particular, if the beamlets are assumed to have a Gaussian profile, two images are needed to analytically extract the amplitude and centre of each beamlet [32]. Furthermore, if a variation in the beamlet width is also considered as a result of small angle scattering, the acquisition of a third image is needed to assess this additional change [33]. The process of disentangling the absorption, refraction and small angle scattering signals from raw acquired images is referred to as phase retrieval. Remarkably, when multi-image acquisition is not feasible, e.g. because of constraints on scanning time or delivered dose, a single image retrieval is also available which provides good image quality [34], [35]. EI has a high degree of flexibility, as it allows the imaging setups to be tailored to the available X-ray source or specific imaging tasks by modifying the system and/or mask geometry [23], [36]. The most interesting capability provided by EI for the purpose of this work is multi-resolution. In EI, spatial resolution is driven by the fact that the specimen is sampled by discrete beamlets, and affected by any additional effects due to pixel cross-talk (due to e.g. signal diffusion in the scintillator if indirect detectors are used). The first point is addressed through the acquisition and re-combination of multiple images acquired while displacing the sample in sub-pixel steps, to cover the missing portions behind the mask septa ("dithered" acquisition). Where cross-talk is significant, this is addressed by using a mask that illuminates every other pixel column (or row), referred to as a skipped mask design (Fig.1(b)). This design reduces the signal diffusion into the next used pixel [24]; more than one column (row) can be skipped where cross-talk is very significant. The combination of skipped masks and dithered acquisitions can bring the ultimate image resolution down to the mask aperture size, even when focal spot and detector resolution are much larger [22]. However, the increase in resolution comes at expense of a longer scanning time and dose delivered to the sample since more images are needed.

### System design

The high resolution system used in this work is based on an EI system originally designed for intra-operative applications [7], [37]. The two systems share the same X-ray source and detector, but employ different masks (see the schemes in Fig.1). The source is a Rigaku 007 rotating Mo anode operated at a constant power of 800 W (40 kV, 20 mA); it features a spot size of about 70 *μm* full width at half maximum (FWHM) [23]. The detector is a Hamamatsu C9732DK flat panel with a pixel size of 50×50 *μm*^2^. The overall system length is 70 cm while sample to detector distance is 15 cm. This arrangement provides a system magnification of 1.25×. Two sets of masks have been designed for the system. The non-skipped detector (sample) mask, already used for intra-operative imaging, has a pitch of 48 *μm* (38 *μm*) and aperture size of 20 *μm* (12 *μm*). In the skipped set, the detector (sample) mask has a pitch of 98 *μm* (78 *μm*) and aperture size of 20 *μm* (12 *μm*). The masks have been manufactured by Microworks GmbH (Karlshrue, Germany) and are made by gold electroplating on a 400 *μm* thick graphite substrate. The thickness of gold layer is > 120*μm* from specifications. Switching between the two combination requires manually replacing the set of masks in use with the new one. Mask alignment has been performed with sub-micron accuracy after each change by means of an automated process [29].

### Image acquisition and processing

To characterize the system resolution when using masks for both low- and high-resolution, a tungsten edge placed at sample position has been imaged. When the non-skipped masks are used, a single 20 second exposure was taken. When the skipped masks are used, images at 8 sub-pixel (dithering) steps were acquired and recombined into the final projection. In both cases, sample and detector masks were aligned with respect to each other. All the images have been corrected by dark subtraction and flat normalization. Edge Spread Functions (ESFs) were then extracted according to the slanted edge method [38]. To characterize system resolution in both cases, the line spread function (LSF) was obtained as the derivative of the (smoothed) experimental measured ESFs.

CT scans of breast specimens have been acquired in both low and high-resolution mode for comparison. For the low resolution scans (intra-operative mode), the non-skipped masks set has been used acquiring 2542 projections over 360 degrees with an exposure time of 1.44 seconds each. The total scanning time was about 1 hour; however, we note this was reduced to 10 minutes in a subsequent version of the prototype developed by our industrial collaborators [7]. Since continuous sample rotation prevented the use of sample “jitter”, a ring artefact suppression algorithm was applied [39]. For the high-resolution CT scans with the skipped masks, the same number of projections were acquired but with an exposure time of 2 seconds each, to compensate for the lower statistic observed when skipped masks are used. In addition, 8 dithering steps for each projection angle have been acquired translating the sample of sub-pixel step of 10 *μm*, for an approximately 10 hours total acquisition time. It is worth noting that the actual scanning time can be longer, because a fly-scan have not been used in this case, which leads to dead times being introduced after each sample movement. While a (partial) fly-scan could be implemented by performing a full sample rotation at each dithering step, at the moment this was prevented by the use of sample “jitter” (translation of the sample of an integer number of the sample mask period) to minimise ring artefacts. For this reason, we do not expect the same 6-fold reduction as observed in the intra-operative case when the high-resolution acquisition modality is implemented with the pre-commercial system [7]; however, a reduction to approximately 2-2.5 hours should be within reach. We are currently working on solutions to avoid jitter altogether, which would enable faster acquisitions based on multiple flyscans along the lines described above; another option to access even faster scans is provided by the recently introduced “cycloidal CT” concept [40].

For both low- and high-resolution scans a single image phase retrieval algorithm was used [34]. This provides the integrated phase (or absorption) coefficient across the sample thickness from a single projection under specific assumptions. It requires the refraction angle to be small enough that a linear approximation of the IC can be used, and the sample to be homogeneous. Under these assumptions, the *β* and *δ* coefficients can be considered proportional, i.e. they can be reduced to a single coefficient *γ*(*E*) = *δ*(*E*)/*β*(*E*). While the first assumption is verified by a large number of specimens, the latter is strictly true only for single material specimens. However, even in cases where this condition is not strictly verified, single image retrieval still provides high image quality with low noise, at the expense of quantitative significance [28], [41]. Under the above assumptions, a retrieved image can be obtained as:

(3) $$\int \mu (x,y,z)dz=-log\left[F^{-1}\left\{\frac{F\{I(x,y)/I_{0}\}}{1-2\pi iz_{od}IC(s_{i})/IC^{\prime }(s_{i})\gamma f_{x}}\right\}\right]$$

where *I*(*x, y*) is the image acquired at sample mask position *s_i_*, *F* denotes the Fourier transform, *IC*′ is the first derivative of the IC and *f_x_* is the spatial frequency along *x.* Scans presented in this work have been acquired at the maximum slope position located at *s_i_* = 9*μm* from the top of the IC, with the latter corresponding to the position for which the masks are aligned. A value of *γ* = 0.7 cm^−1^ (corresponding to 19 keV, compatible with the average energy of the used molybdenum spectrum) was used. The retrieval algorithm was applied to each individual projection after normalization and before CT reconstruction, which was performed assuming cone beam geometry. It is worth noting that the low- and high-resolution scans were performed several weeks apart, with the specimens being stored in a fridge in the intervening time. Therefore, some degree of tissue shrinkage may have happened in between the two scans. In addition, the sample has been repositioned in the holder, which required a manual selection of the best matched resliced volume slice for both scans. Both these effects may have led to a non-perfect match between the low- and high- resolution images.

### Sample preparation

Breast wide local excision specimens were collected fresh from surgery following informed patient consent for use of tissues in research (BCI TB NRES Approval from East of England, Central Cambridge N RES 15/EE/0192). The samples were approximately 30 mm superior to inferior, 15-20 mm medial to lateral and 10 mm anterior to posterior, typical of many breast samples. The samples were inked and weighed, according to standard protocols, then fixed in 10% formal saline for 24h before the X-ray scans. During the CT scans the samples have been placed into plastic pots without further processing. Following imaging, histological examination was carried out on all specimens. The entire specimen was blocked out for analysis, taking full-face slices into conventional or megablock cassettes. These underwent routine processing and embedding in paraffin wax. A 4 *μ*m section was taken from each block and stained with H&E according to standard protocols.

## Supplementary Material

supp1-3137964
