# Supplementary material for: Volumetric high-resolution X-ray phase-contrast virtual histology of breast specimens with a compact laboratory system
Source: IEEE Trans Med Imaging. Author manuscript; Available in PMC 2022 May 21. (PMC7612751; doi:10.1109/TMI.2021.3137964)
Supplement: supp1-3137964 [file EMS144691-supplement-supp1_3137964.pdf]

Volumetric high-resolution X-ray phase-contrast virtual histology of breast specimens with a compact laboratory system: supplementary material

Lorenzo Massimi[a], Tamara Suaris [b], Charlotte K. Hagen[a], Marco Endrizzi[a], Peter R. T. Munro[a], Glafkos Havariyoun[a], P. M. Sam Hawker [c], Bennie Smit [c], Alberto Astolfo [c], Oliver J. Larkin [c], Richard M. Waltham [c], Zoheb Shah [d], Stephen W. Duffy [d], Rachel L. Nelan [d], Anthony Peel [b], J. Louise Jones [b] [d], Ian G. Haig [c], David Bate [c], Alessandro Olivo[a]

[a] Department of Medical Physics and Biomedical Engineering, University College London, London, UK

[b] St Bartholomew's Hospital, Barts Health NHS Trust, West Smithfields, London, UK

[c] Nikon X-Tek Systems, Tring Business Centre, Icknield Way, Tring, Hertfordshire, UK

[d] Barts and the London School of Medicine and Dentistry, Queen Mary University of London, Newark St, London, UK

Effects of paraffin embedding

As standard operation in histological workflow breast specimens are usually embedded in wax before slicing. The wax embedding process is known to shrink tissues altering their appearance compared to X-ray phase contrast tomography. This effect may change the depth of a suspect lesions identified by virtual histology preventing further investigation of the same features with

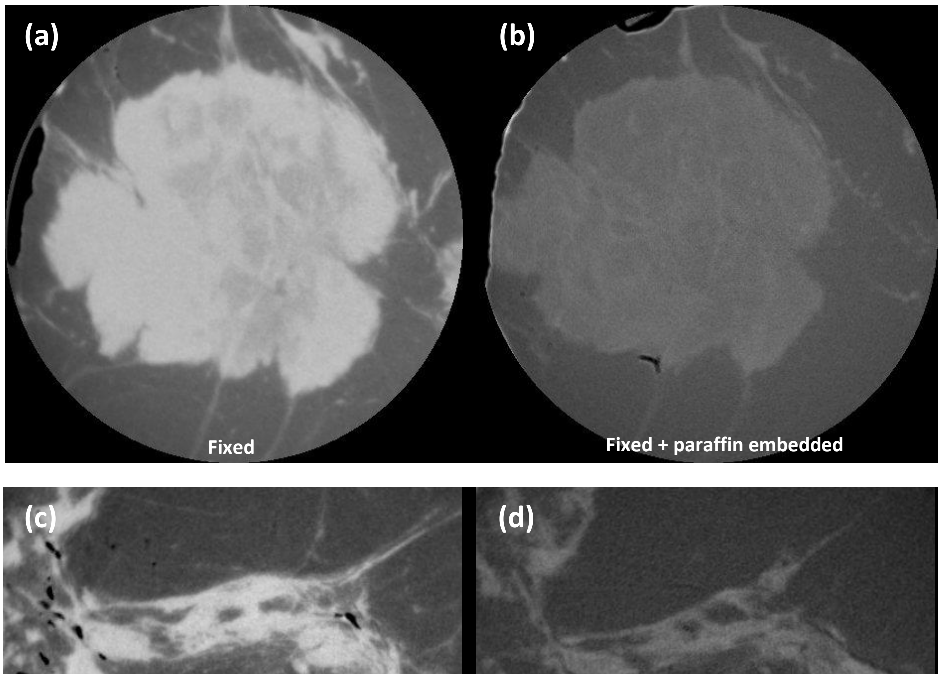

Figure S1 Effect of wax embedding. Panels (a) and (c) show CT slices for two different breast specimens after fixation. Panels (b) and (d) shows the corresponding CT slices (calculated from the top of the specimen) for the same samples after paraffin embedding.

histology. To test the impact on breast tissues of was embedding, we scanned two breast specimens before and after wax embedding. The CT slices corresponding to the sample depth, measured from the top of the specimens, are shown in Fig.S1. The first evident effect is the decrease of contrast between fibroglandular and fat tissues, because of wax embedding found in both samples. However, a visual inspection of both samples reveals that the sample aspect has not changed, and no significative shrinking occurred. This measurement makes us confident that, if a suspect lesion is found with X-ray, is position will not be significantly altered by wax embedding making possible to find the same location for tissue slicing for further analysis.

Tumour bed segmentation

In the main manuscript the identification of low density region withing the fibroglandular tissue has been highlighted. Through histology these regions have been identified as areas associated with inflammatory response which is typical of response to chemotherapy treatments. Therefore, segmentation of these regions allows their quantification and opens to the possibility to assess the response to treatments through phase contrast CT. Two examples of segmentation performed through Weka Trainable [1] pixel classification algorithm provided in imageJ are reported in Fig. S2.

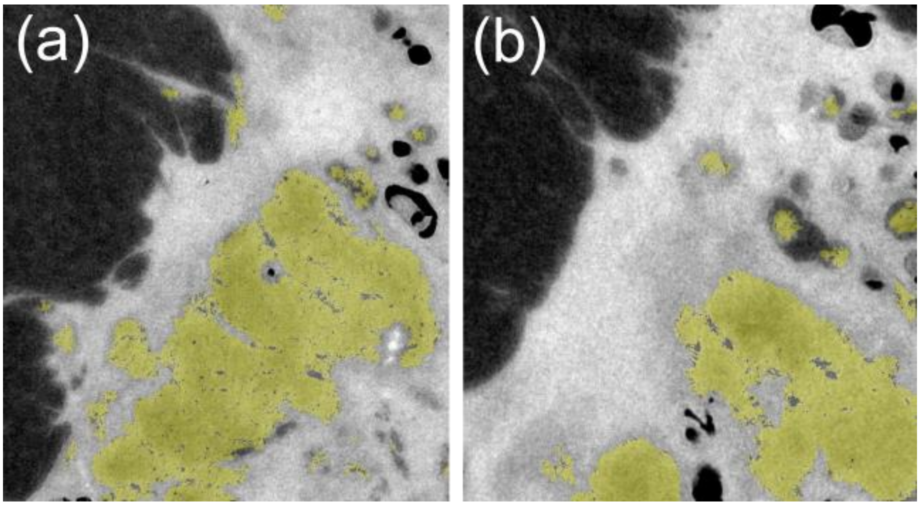

Figure S2. Segmentation of tumour bed (yellow area(in two different CT slices

[1] Arganda-Carreras, Ignacio, et al. "Trainable Weka Segmentation: a machine learning tool for microscopy pixel classification." *Bioinformatics* 33.15 (2017): 2424-2426.
